# Zearalenone Removal from Corn Oil by an Enzymatic Strategy

**DOI:** 10.3390/toxins12020117

**Published:** 2020-02-13

**Authors:** Xiaojiao Chang, Hujun Liu, Jing Sun, Jun Wang, Chengcheng Zhao, Wan Zhang, Jie Zhang, Changpo Sun

**Affiliations:** 1 Institute of Grain & Oil Science and Technology, Academy of National Food and Strategic Reserves Administration, Beijing 100037, China; cxj@chinagrain.org (X.C.); lhj@chinagrain.org (H.L.); sj@chinagrain.org (J.S.); wj@chinagrain.org (J.W.); zcc@chinagrain.org (C.Z.); zhangwan16@mails.jlu.edu.cn (W.Z.); 2 Institute of Plant Protection, Chinese Academy of Agricultural Science, Beijing 100094, China; zhangjie05@caas.cn

**Keywords:** zearalenone, enzymatic reaction, detoxification, corn oil, refining

## Abstract

The estrogen-like mycotoxin zearalenone (ZEN) is one of the most widely distributed contaminants especially in maize and its commodities, such as corn oil. ZEN degrading enzymes possess the potential for counteracting the negative effect of ZEN and its associated high safety risk in corn oil. Herein, we targeted enhancing the secretion of ZEN degrading enzyme by *Pichia pastoris* through constructing an expression plasmid containing three optimized expression cassettes of *zlhy-6* codon and signal peptides. Further, we explored various parameters of enzymatic detoxification in neutralized oil and analyzed tocopherols and sterols losses in the corn oil. In addition, the distribution of degraded products was demonstrated as well by Agilent 6510 Quadrupole Time-of-Flight mass spectrometry. *P. pastoris* GSZ with the glucoamylase signal was observed with the highest ZLHY-6 secretion yield of 0.39 mg/mL. During the refining of corn oil, ZEN in the crude oil was reduced from 1257.3 to 13 µg/kg (3.69% residual) after neutralization and enzymatic detoxification. Compared with the neutralized oil, no significant difference in the total tocopherols and sterols contents was detected after enzymatic detoxification. Finally, the degraded products were found to be entirely eliminated by washing. This study presents an enzymatic strategy for efficient and safe ZEN removal with relatively low nutrient loss, which provides an important basis for further application of enzymatic ZEN elimination in the industrial process of corn oil production.

## 1. Introduction

Zearalenone (ZEN), the secondary metabolite of *Fusarium* species that causes contamination through the food chain, has attracted particular attention worldwide. Its genotoxicity, carcinogenicity, immunotoxicity, teratogenicity, hepatotoxicity, hematotoxicity, and the strong estrogenic activity are all deleterious to animals and humans. For example, ZEN disturbs hormonal homeostasis and induces numerous diseases [1,2,3,4]. ZEN is frequently detected in various grains and their agricultural commodities, especially in maize [5]. The contamination occurs in the field and during the period of planting, harvesting, storage, transportation, and processing when exposed to the appropriate environmental conditions [6,7,8]. The commonly consumed corn oil contains high nutritional contents of vitamin E, plant sterols, and polyunsaturated fatty acids [9]. During the industrial pressing process, maize gum is expected to be prone to fungal infection and apolar ZEN contamination in corn oil. The concentration of ZEN from contaminated corn oil in reported outbreaks was up to 1730 μg/kg [10,11,12,13]. As the awareness and understanding of the increased ZEN exposure-associated risks, the European Commission (EC) has established and enforced a maximum 400 µg/kg ZEN level in refining corn oil, and the tolerable daily intake (TDI) of ZEN has been set at 0.25 µg/kg b.w. per day based on the collected toxicity assessment and exposure data [14]. Moreover, the national regulation on the limitation of ZEN in such parameters has also been revised constantly in China.

Various strategies including mycotoxins mitigation, physical absorption, chemical detoxification, and biological degradation have been applied in food commodity. Several previous studies have also reported such strategies utilized in edible oil primarily through physical and chemical approaches. For instance, Bai et al. [15] reported an adsorption strategy in detoxification of ZEN from corn oil by developing a functionalized graphene oxide material (FGO) using amphiphilic molecules didodecyldimethyl-ammonium bromide (DDAB) as the modifier; Mao et al. [16] used ultraviolet irradiation to detoxify aflatoxins in peanut oil and analyzed the structures and toxicity of the degraded products. Several researchers have found that mycotoxins, such as ZEN, aflatoxin B_1_, and deoxynivalenol, in edible oil could be detoxified during the alkali refining [11,17]. Though physical adsorption strategy could remove ZEN efficiently, it results in a great deal of nutrient loss including vitamin E and sterols due to the non-specificity in the process of adsorption [18,19]. Chemical detoxification as well, is an efficient method in breaking the structure of ZEN, but it is associated with several limitations especially the disruption of the palatability and nutritional compositions in edible oil by strong alkalis or oxidants [16]. Furthermore, the detoxified products still remained in the oil which may potentially cause other complex chemical reactions inducing further toxicity.

**Figure 1:**
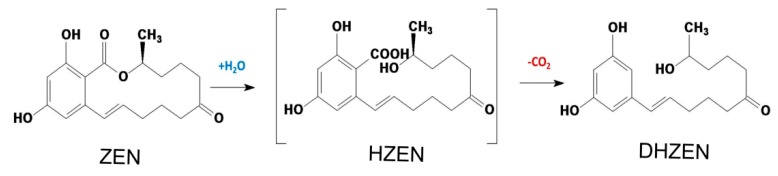
Mechanism of zearalenone (ZEN) degraded into hydrolyzed zearalenone (HZEN) and decarboxylated hydrolyzed zearalenone (DHZEN) by lactonohydrolase ZHD101 (Adapted from [25]).

Biotechnology possesses the largest potential for future developments of biological strategies in food industry [20,21]. As a matter of fact, enzymes from microbial sources are more efficient transforming and degrading ZEN naturally present in food commodities, and they can also be applied extensively in food products processing [20]. *Clonostachys rosea* (synonym of *Gliocladium roseum*) was documented with its capability to metabolize the ester bond in the lactone ring of ZEN in high yield into product with less estrogenic activity [22]. Subsequently, the lactonohydrolase ZHD101 from *C. rosea* catalyzing this reaction was characterized and its encoding gene was found and cloned [23]. Further, the reaction mechanism was also unveiled [24] and the structures of degradation products were confirmed as well, which were followed by evaluation on the toxicity of these products (Figure 1) including hydrolyzed ZEN (HZEN) and decarboxylated hydrolyzed ZEN (DHZEN) [25].

Therefore, the study presented here utilized the ZEN degrading enzymes ZLHY-6 as an example to investigate the feasibility of enzymatic removal of ZEN from corn oil during the alkali refining process on the basis of optimal expression of ZLHY-6. Furthermore, we also aim to analyze the distribution of the degraded products and the losses of the total tocopherols and sterols in the corn oil during this enzymatic reaction. The underling enzymatic removal of ZEN in corn oil might provide a new avenue for the detoxification of mycotoxin both efficiently and safely from vegetable oil through biological enzyme catalysis.

## 2. Results

### 2.1. Construction of ZEN Degrading Enzyme

The amino acid sequence of ZLHY-6 showed high identity (98.3%) with ZHD101 (zearalenone hydrolase) with the putative catalytic triplet SHE motif (Ser105-His243-Glu129). With optimization, a total of 172 base pairs, involving 97 codons, of *zlhy-6* were replaced according to the codon usage preference of *Pichia pastoris*. The codon adaptation index (CAI) was increased from 0.069 to 0.717 and the GC content was reduced from 56.0% to 46.7%. Further, the stability of mRNA and the structure of stem-loop was also confirmed. The codon-optimized version of the gene was submitted to GenBank (Accession No. MN933397).

**Figure 2:**
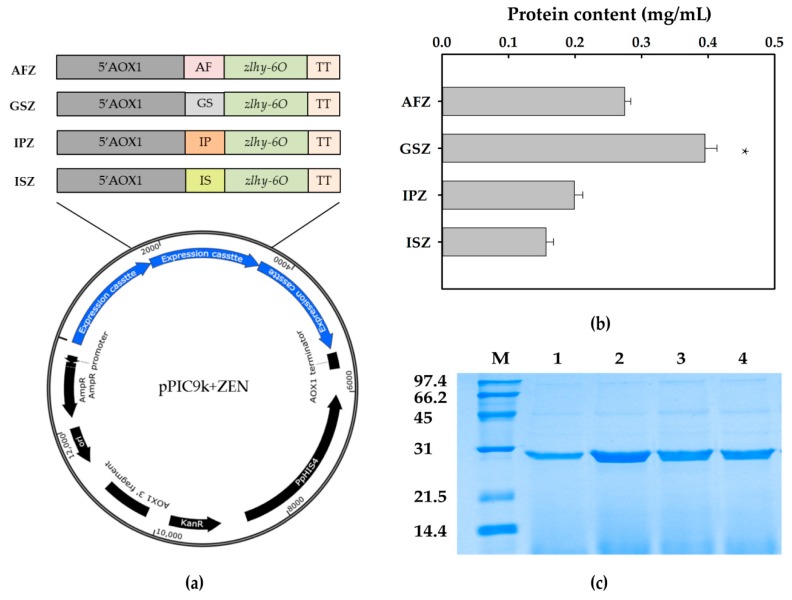
The schematic diagram of the four recombinants and the comparison of secretion expression levels of ZLHY-6. (**a**) Schematic diagram of the four constructed recombinant plasmids containing three copies of *zlhy-6O* expression cassettes led by an individual signal peptide. (**b**) The comparison of the secretion yields of ZLHY-6 induced by four different signal peptides. Each result represents the mean ± SD with three replicates per experiment. Significant differences are indicated with * (*p* < 0.05). (**c**) SDS-PAGE analysis of the four recombinants induced by different signal peptides. Lane M: protein ladder; lanes 1–4, fermented supernatants from recombinant *Pichia pastoris* with 96 h induction by AFZ, GSZ, IPZ, and ISZ, respectively.

The expression cassette of the optimized gene *zlhy-6O* with three copies were constructed into pPIC9k vector with different secretory components including α-mating factor (AF), glucoamylase signal sequence (GS), inulinase presequence (IP), and invertase signal sequence (IS) through the Biobrick method, separately (Figure 2a). The *Sal* I-linearized products of the four recombinant plasmids were transformed into GS115, selected by MD medium, and sequenced by PCR consecutively. The target recombinants with three copies of *zlhy-6O* expression cassettes were named as AFZ, GSZ, IPZ, and ISZ, respectively.

### 2.2. Expression of ZEN Degrading Enzyme

The supernatants from the fermented cultural liquids were collected every 24 h and analyzed by SDS-PAGE. The band of the target protein was consistent with its theoretical size of approximately 28 kDa. Among ZEN degrading enzyme expression in the four recombinants, *P. pastoris* GSZ guided by glucoamylase signal was detected with the highest expression level. The protein determination showed the expression yield of AFZ, GSZ, IPZ, and ISZ were 0.27, 0.39, 0.20, and 0.16 mg/mL, respectively (Figure 2b). According to the SDS-PAGE analysis, most proteins detected in the fermented supernatants were the ZEN degrading enzyme (Figure 2c). Therefore, it provided us the confidence to further evaluate the fermented supernatants for corn oil detoxification directly without purification in this study.

### 2.3. Preparation of ZEN Degrading Enzyme

Approximately 1.5 L of fermented supernatant was collected from five 2 L flasks, in which GSZ with the glucoamylase signal was cultivated and induced by 1.5% (v/v) methanol every 24 h (data not shown). The Image Lab Version 5.2.1 software was used to quantitatively analyze the content of ZLHY-6 through the gray-scale value of the target band in SDS-PAGE, and it was found that the content of target protein accounted for 92.3% of the total protein. Therefore, approximately 400 mL of ZEN degrading enzyme solution at the concentration of 1.5 mg/mL determined by the Bicinchoninic Acid Protein Assay Kit was concentrated using an ultrafiltration centrifuge tube. 

### 2.4. Detoxification of ZEN in Corn Oil by the Degrading Enzyme

**Figure 3:**
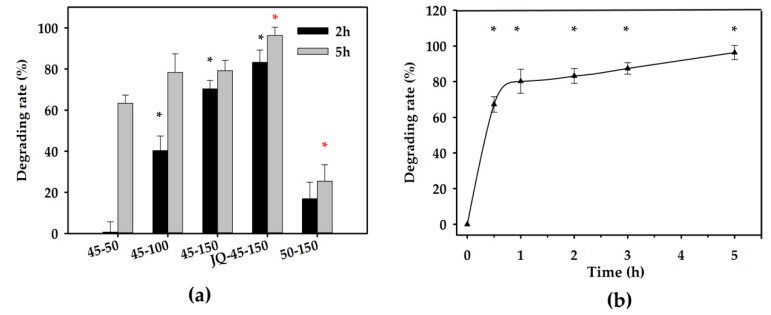
ZEN degradation in corn oil under different treatments. (**a**) Corn oil treated for 2 and 5 h by 50 mg/kg ZLHY-6 at 45 °C, 100 mg/kg ZLHY-6 at 45 °C, 150 mg/kg ZLHY-6 at 45 °C, 150 mg/kg ZLHY-6 at 45 °C with 1 min of pre-homogenization, 150 mg/kg ZLHY-6 at 50 °C with 100 rpm gentle vortex, respectively. (**b**) Pattern of 0–5 h ZEN degradation with 150 mg/kg ZLHY-6 and gentle vortex after 1 min of pre-homogenization in 3 L bioreactor. Each result represents the mean ± SD with three replicates per experiment. Significant differences are indicated with * (*p* < 0.05).

The effects of the amount of degrading enzyme, reaction temperature, reaction time period, and the mixing strength on ZEN degradation performance in corn oil were investigated in this study. The concentration of ZEN in crude corn oil before and after neutralization was 1257.30 and 617.45 µg/kg, respectively. It was clearly indicated that ZEN in corn oil was degraded (Figure 3). The efficiency of enzymatic degradation increased with extension of the reaction time and enzyme when incubated at 40 (data not shown) and 45 °C at 220 rpm. However, the enzymatic degradation efficiency on ZEN was found to decrease at 50 °C due to the heat inactivation of the degrading enzyme (Figure 3a). Compared with gentle vortex, high shear mixing for 1 min before incubation was associated with more intensive degradation. To be specific, the degrading rate with high shear mixing was approximately 96.31%, and the ZEN remaining concentration after 5 h enzymatic reaction was 13.00 µg/kg (Figure 3a). Moreover, to demonstrate the degradation pattern of ZEN in neutralized corn oil with homogenization, a further study was performed on different incubation time periods. ZEN significantly decreased (degrading rate of 75.3% and ZEN residue of 152.40 µg/kg) with 0.5 h incubation. Whereas, with the extension of incubation time period, the degradation efficiency improved continuously in a time-dependent manner and only 3.69% ZEN remaining in corn oil was detected after enzymatic reaction for 5 h (Figure 3b).

### 2.5. Distribution of Degradation Products

**Figure 4:**
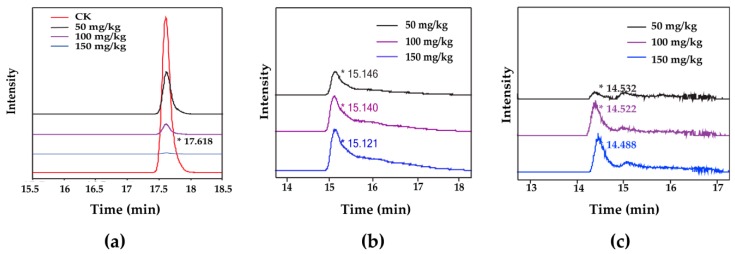
The ion chromatograms for intensity of ZEN and the degradation products (HZEN, DHZEN) in corn oil detoxified with different concentrations (50–150 mg/kg) of degrading enzyme. (**a**) ZEN. “CK” means the neutralized oil treated with the same amount of water without enzyme. (**b**) HZEN. (**c**) DHZEN.

To examine the enzymatic removal of ZEN, ZEN, HZEN, and DHZEN in the oil layer and the water layer from each sample were measured by HPLC-MS/MS. With 5 h incubation, ZEN (retention time of 17.618 min) was degraded effectively by the ZEN degrading enzyme at concentrations from 50 to 150 mg/kg (Figure 4a), leading to the formation of two products HZEN with the approximate retention time of 15.1 min (Figure 4b) and DHZEN with the approximate retention time of 14.5 min (Figure 4c).

**Figure 5:**
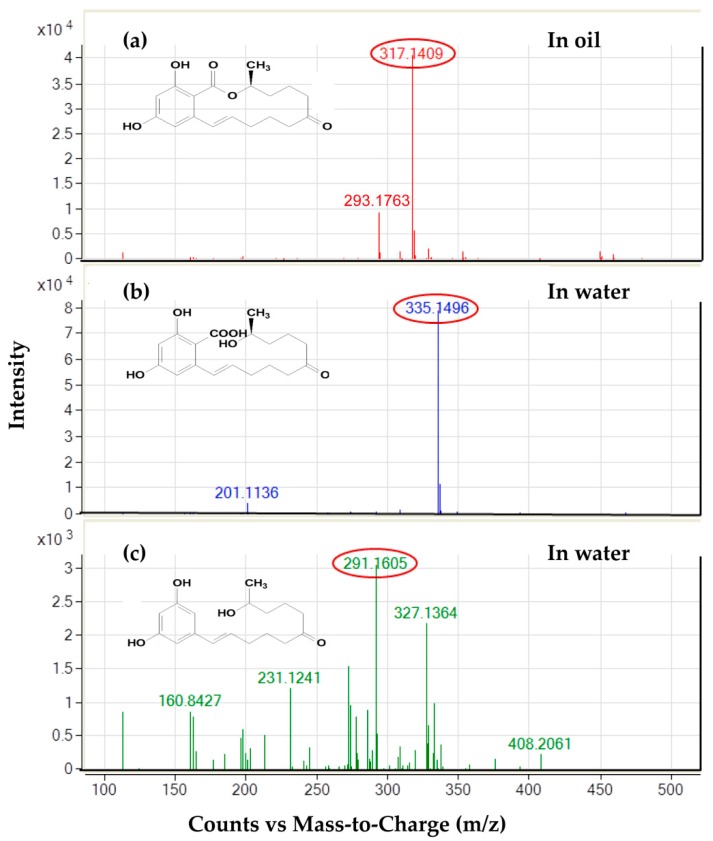
The distribution intensity analysis of ZEN and its products (HZEN and DHZEN) in water or corn oil by LC-MS. (**a**) ZEN in corn oil. (**b**) HZEN in water. (**c**) DHZEN in water.

To examine the distribution of degradation products, the base signals at m/z 335, 291, and 317 amu under full scan measurements (enhanced mass scan, EMS mode) were presented for ZEN and its two chromatographically thoroughly separated products, respectively. The outcomes were consistent with the deprotonated molecular ions [M-H]^−^ for the structures of the three compounds mentioned above. According to the electrospray ionization (ESI) spectrum in negative ion mode, the deprotonated molecular ion [M-H]^−^ of apolar ZEN at m/z 317 amu existed only in the oil layer, as expected (Figure 5a). On the other hand, the signals for polar HZEN and DHZEN at m/z 335 and 291 amu, separately, were detected in the water layer extraction, which were removed by centrifugation at 4000 rpm for 10 min after enzymatic hydrolysis (Figure 5b,c).

### 2.6. Analysis of Tocopherol and Sterol Contents

**Figure 6:**
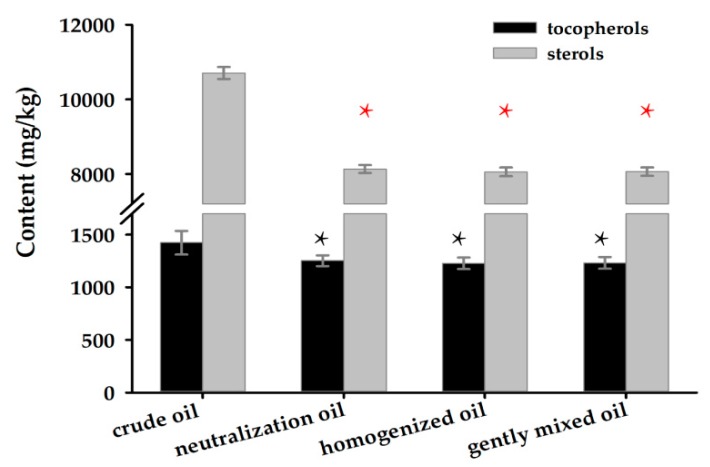
The analysis of tocopherol and sterols contents in corn oil with different experiments. Each result represents the mean ± SD with three replicates per experiment. The black * concerns the comparison on tocopherols in crude oil, and the red * concerns the comparison on sterols (*p* < 0.05).

The alterations of natural tocopherol homologs (α, β-γ, and δ isomers) and total sterol content in crude oil, neutralized oil, and enzymatically detoxified oil were investigated. The total concentrations of tocopherols and sterols in crude corn oil were 1423.17 and 10,705.60 mg/kg, respectively (Figure 6). However, the contents of these two compounds were found to be reduced by 12.09% and 24.03%, respectively, after neutralization reaction during oil refining. No significant (*p* < 0.05) changes in the concentration of these two nutrients were observed among detoxified oils and neutralized oil.

## 3. Discussion

Although physical and chemical approaches offer a certain extent of efficient reduction on such mycotoxin in food products at the post-harvest level currently, their disadvantages in lower specificity, inducing nutrient loss, higher energy consumption, and further safety issues in food commodities cannot be ignored [20]. Hence, the biotechnology approach exhibits the highest potential on minimizing the negative effects of mycotoxins for future developments [20]. From a technological point of view, due to the distinguished feature of higher specificity and efficiency, enzymatic catalysis takes a unique position in transforming mycotoxins into products with less toxicity, which ensures the minimum contamination and safety in commodities destined for human food and animal feeds. Moreover, sufficient studies carried out on lactonohydrolase, such as ZHD101, ZLHY-6, ZHD518, ZHDC, and ZHDG, have reported their capabilities to degrade ZEN and confirmed the chemical structures and toxicity of the metabolites [26,27,28]. Therefore, the present study aimed to evaluate ZLHY-6 as an example to explore the feasibility of removing ZEN from corn oil through enzymatic catalysis.

The yield of heterologous expression plays a crucial role to ensure the effectiveness and application of the target enzyme. As a matter of fact, *P. pastoris* has been successfully established as an important expression host for enzyme production. Furthermore, several factors such as codon usage, secretion signals, and the dosage of the target gene may all have significant impacts on the yield of heterologous protein expressed in *P. pastoris* [29]. Previously, the highest yields of ZHD101 obtained from a *P. pastoris* X3c strain harboring three expression cassettes were found as 22.5 U/mL in a shake flask and 150.1 U/mL in a 5 L fermenter [30]. Likewise, we constructed the recombinants in GS115 with three expression cassettes of *zlhy-6* with optimized codons. Signal peptides possessing relatively high secretory capacity were selected as inducers, which included α-mating factor and invertase signal sequence derived from *Saccharomyces cerevisiae*, glucoamylase signal sequence derived from *Aspergillus awamori*, as well as inulinase presequence derived from *Kluyveromyces maxianus* [29]. As a result, the concentration of ZLHY-6 obtained from the fermented supernatant reached up to 0.39 mg/mL, and the enzymatic activity was detected as 181 U/mL in *P. pastoris* which was much higher than the one previously reported. However, from the point of industrial application, a significant promotion in enzyme expression yield is warranted for larger fermentation scale. Industrial application costs will also be reduced with the increasing of the yield of degrading enzyme expression. Since *G. roseum NRRL 1859* was reported for its capability to cleave the ester bond in the macrolactone of ZEN from 1988 [31], multiple studies were carried out focusing on the isolation and characterization of lactonohydrolase ZHD101, and the degradation mechanisms as well as the toxicity of the degraded products have also been reported during the following three decades [23,25,32,33,34]. Later on, the structures of ZHD101-ZEN and ZHD101-α-ZOL (the derivative of ZEN with 92-fold higher estrogenicity) complexes were further determined and analyzed [35]. The mutant V153H of ZHD was constructed, which maintained its activity against ZEN but exhibited 3.7-fold increase in its specific activity against α-ZOL [36,37,38,39]. All studies mentioned above provide a solid foundation for further industrial applications of ZHD101. On the other hand, ZLHY-6 was demonstrated possessing a high identity (98.3%) with ZHD101 and with similar enzymatic properties. The optimal temperature and pH for ZLHY-6 towards ZEN were observed ranging from 25 to 41 °C and 6.5 to 10, respectively [40]. Moreover, during the industrial process of alkaline refining, phospholipid removal through neutralization by sodium hydroxide is required for refining corn oil [41], and we have determined the pH of the neutralized corn oil as 6.4. A great amount loss of enzymatic activity at 45 °C in water was observed as expected (data not shown). Whereas, ZLHY-6 exhibited higher tolerance in corn oil compared with that in water at the same temperature and pH, further studies on ZLHY-6 hydrolysis in oil and water are essential for revealing the mechanism of lipases. In addition, the development of ZEN degrading enzyme with higher temperature-tolerance through computational biology is necessary as well for improving the function and efficiency in industrial ZEN removal.

The maximum level of ZEN allowed in refined corn oil defined by European Union (EU) is 400 μg/kg [14], and the provisional maximum tolerable daily intake (PMTDI) of ZEN by the Joint Expert Committee on Food Additives (JECFA) is implemented as 0.5 μg/kg body weight [42]. Further, the post-safety of mycotoxin-contaminated edible oil after detoxification is also a crucial factor for evaluating the feasibility of ZEN removal strategies. During the neutralization of alkali refining, ZEN was significantly reduced due to its phenolic hydroxyl group being neutralized by alkali [17]. In the present study, ZEN in crude corn oil (1257.30 µg/kg) was deducted by 50.89% after neutralization, which was consistent with previous report [20]. However, we also observed that the alteration of ZEN described above was reversible, which will make the corn oil under high risks. Due to the approximate 100% ZEN recovery rate at low temperature, it is also important to control a high temperature environment for ZEN removal during the neutralization process. In this study, the irreversible ZEN degradation was achieved at a higher rate of 96.31% (13 µg/kg) through enzymatic catalysis (Figure 3). Moreover, HZEN and DHZEN have been proven to have 50–10,000 fold lower estrogenicity compared with ZEN in vitro, and that no impact was found in vivo on vulva size or uterus weight in piglets, though alterations at the gene transcriptional level were observed [25]. Overall, ZEN detoxification in corn oil achieved through enzymatic catalysis was proven to be both efficient and safe.

Finally, to be noted, consecutive steps including degumming, neutralization, bleaching, deodorization, and winterization are conducted for refining crude oil [43]. During these processes, not only the undesirable compounds such as free fatty acids, waxes, polar lipids, oxidation products, metal ions, and pigments were excluded but also several desirable nutrients like antioxidants and triglycerides could be removed as well [44]. In addition, excessive alkaline treatment combined with high temperature is needed during oil neutralization and deodorization processes for mitigating ZEN contamination, which normally causes a major loss of neutral oil in refining corn oil [9,15,17,20]. Furthermore, abundant natural tocopherols and sterols with antioxidant activity present in corn oil could easily be removed through deodorizer distillates at high temperature [45]. Therefore, conventional physicochemical approaches not only have the limitation on ZEN removal from corn oil, but also reduce the oil production yield, increase energy consumption, and lead to the unnecessary loss of main functional components such as tocopherols and sterols.

In comparison, a physical detoxification strategy was performed by using functionalized graphene oxide to remove ZEN from corn oil by π–π interaction and hydrogen bonding by using sorbents not only cannot ensure the safety of the material, but also a large amount of losses of tocopherols and sterols caused by the non-specific adsorption [15]. The alkali refining strategy to remove ZEN was conducted by adding excessive alkali and increasing the refining temperature, which results in high energy consumption, reduced oil yield, and the losses of functional components. Moreover, most ZEN could be recoverable when the simulated alkali refining systems returned to neutral or at low temperature of 30 °C [17]. However, the enzymatic strategy is safe and high efficient on ZEN removal from corn oil with relatively low nutrient loss.

## 4. Conclusions

To our knowledge, this is the first report to use enzymes in the removal of ZEN from corn oil during oil production. Enhanced secretion of ZEN degrading enzyme by *P. pastoris* GSZ was conducted by constructing three expression cassettes of *zlhy-6* with optimized codons and by glucoamylase signal induction. It demonstrated that ZEN in the crude oil with an initial concentration of 1257.3 µg/kg was remarkably reduced to 13 µg/kg through neutralization and enzymatic detoxification. The end products of ZEN, HZEN, and DHZEN, were extracted and analyzed by Agilent 6510 Quadrupole Time-of-Flight LC/MS. It was found that these two degradation products were transferred and eliminated by washing. Moreover, no obvious change in the contents of total tocopherols and sterols after enzymatic detoxification was detected when compared with the neutralized oil. The present findings provide an important reference for safe ZEN removal during industrial corn oil production.

## 5. Materials and Methods 

### 5.1. Plasmids, Strains, Chemicals, and Reagents

*Escherichia coli* DH5α from Stratagene (Agilent Technologies, Santa Clara, CA, USA) was used as the cloning host strain. The plasmid pPIC9k and *Pichia pastoris* GS115 from Invitrogen (Invitrogen, Carlsbad, MA, USA) were used for gene expression. Yeast extract peptone dextrose (YPD) medium, buffered complex glycerol (BMGY) medium, buffered complex methanol (BMMY) medium, and minimal dextrose (MD) medium were prepared according to the instruction of Pichia Expression Kit (Invitrogen, Carlsbad, MA, USA).

The standards of zearalenone (ZEN, GSB 11-3429-2017) with > 99.6% purity was purchased from Academy of National Food and Strategic Reserves Administration (Beijing, China). α-, β-, γ-, and σ-tocopherols were purchased from Sigma–Aldrich (St. Louis, MO, USA). The standards of sterols were purchased from Sigma–Aldrich (St. Louis, MO, USA). The crude corn oil was donated by a corn oil production enterprise of China. Restriction enzymes (*Eco*R I, *Not* I, *Bam*H I, *Not* I, and *Sal* I) were purchased from Thermo Fisher (Waltham, MA, USA). T4 DNA ligase and Taq DNA polymerase were purchased from TaKaRa (Dalian, Liaoning, China). Acetonitrile and methanol of HPLC grade were purchased from Thermo Fisher (Waltham, MA, USA). 

### 5.2. Preparation of ZEN Degrading Enzyme

#### 5.2.1. Construction and Screening of the Recombinant Strain

ZEN degrading gene *zlhy-6* was cloned from *C. rosea* 31535 [40] by our laboratory and submitted to GenBank (Accession No. HQ825318.1). To enhance the secretory expression, the cloning was performed as follows: (i) the codon-optimized version of the target gene was synthesized by GenScript (Nanjing, Jiangsu, China) according to *P. pastoris* codon usage preference for reaching the highest protein expression; (ii) four signal peptides were selected from previous reports [46,47,48,49]. The *zlhy-6* optimization sequence *zlhy-6O* was then inserted into pPIC9k with an α-mating factor encoding sequence through *Eco*R I and *Not* I. Further, three signal peptides (glucoamylase signal sequence, inulinase presequence, and invertase signal sequence) were synthesized in front of the 5’-primer targeting region in *zlhy-6O* to replace the α-factor secretion signal through *Bam*H I and *Not* I. Sequences of signal peptides and primers used in this study are shown in Table 1; (iii) three copies of the *zlhy-6O* expression cassettes constructed were reported to exhibit the highest ZHD expression [30], therefore the expression cassettes with three copies of the zlhy-6O were inserted into pPIC9k with the Biobrick method [50] in *E. coli* DH5α. The recombinant plasmid was then transformed into the competent *P. pastoris* GS115 through electroporation (2.2 kV, 25μF, 200Ω) after being linearized by *Sal* I. Several transformants were screened on MD plates and YPD plates. The selected colonies were identified by PCR and sequencing afterwards. The expression yield of the recombinants was analyzed and determined by Bicinchoninic Acid Protein Assay Kit (Sigma-Aldrich, St. Louis, MO, USA).

#### 5.2.2. Expression and Preparation of ZEN Degrading Enzyme

*P. pastoris* transformants were collected and centrifuged after being cultivated in 250 mL shake flask with 50 mL BMGY for 48 h (28 °C, 220 rpm). The expression of ZEN degrading enzyme was performed in a 2 L shake flask with 400 mL BMMY by adding 1.5% (v/v) methanol every 24 h for 96 h. The fermented cell-free cultural supernatant was then collected by centrifugation at 12,000 rpm for 5 min. The expression yield and purification of ZLHY-6 were determined and analyzed by Bicinchoninic Acid Protein Assay Kit and Image Lab Version 5.2.1 software (Bio-Rad Laboratories, Berkeley, CA, USA), respectively. To prepare for the corn oil detoxification assay, ZEN degrading enzyme ZLHY-6 at the concentration of 1.5 mg/mL was concentrated using an ultrafiltration centrifuge tube (Millipore Amicon Ultra-15, 10 kDa, Darmstadt, Germany).

### 5.3. Retreatment of Crude Corn Oil

The simulated degumming and neutralization of the industrial corn oil refining process were performed based on previous methods described by Cheng et al. with slight modifications with the standard procedures of corn oil industrial production [18,51]. Briefly, the simulated process of degumming was carried out by adding 85% phosphoric acid into 1000 g of the crude corn oil at a ratio of 0.1% (v/w), followed by dispersing under high shear mixing (13,000 rpm) for 1 min with homogenizer (IKA^®^ T18 digital ultra turrax, Staufen, Germany). The degumming reaction was conducted at 75 °C for 20 min with gentle mixing. To neutralize the free fatty acid content (FFA), 15 g of 18 Be’ sodium hydroxide (NaOH, 12.64%, w/w) was added into the oil mixture and heated at 85 °C for 20 min in a water bath after dispersing at 13,000 rpm for 1 min. Subsequently, the mixture was centrifuged at 4000 rpm for 10 min and the supernatant was collected and prepared for the enzymatic degradation assay.

### 5.4. Experimental Design and Treatment

#### 5.4.1. Detoxification of ZEN in Corn Oil

An aliquot of 50 g neutralized oil for each sample was weighed in a 100 mL centrifuge tube. A total of 0.17–0.5 mL of ZLHY-6 at the concentration of 1.5 mg/mL (final concentration 50–150 mg/kg) was diluted with sterile water to ensure that the total amount of enzyme mixture reached up to 5 g (10% w/w in the neutralized oil). The enzymatic degradation assays were performed as follows: (I) the mixture was incubated at 40–50 °C with gentle mixing (100 rpm) over 2–5 h; (II) the mixture was dispersed at 13,000 rpm for 1 min before incubation at 40–50 °C with gentle mixing (100 rpm) for 2–5 h; (III) to simulate the corn oil refining process, a larger scale of corn oil detoxification was performed in a 3 L bioreactor (Biostat^®^ D-DCU, Sartorius, Goettingen, Germany). The mixture was dispersed at 13,000 rpm for 1 min before incubation at 45 °C with gentle mixing (100 rpm) for 0.5–5 h. Subsequently, the enzymatic reactions were stopped by centrifugation at 4000 rpm for 10 min to separate the enzyme from the oil mixture.

#### 5.4.2. Extraction and Analysis for ZEN in Corn Oil 

The reaction mixture of each treatment was centrifuged at 4000 rpm for 5 min. The upper oil layer (A) and the lower water layer (B) of each sample were collected and transferred into fresh 50 mL centrifuge tubes for further analysis. 

The extraction and purification of zearalenone in corn oil were determined as previously described by Guo et al. with slight modifications [52]. Briefly, 10 g of corn oil was weighed and mixed with 100 mL of acetonitrile/water (9:1, v/v). Following at 3000 rpm for 5 min, 10 mL of the extract was mixed with 40 mL of phosphate buffer solution tween-20 (PBST, 8 g NaCl, 0.2 g KCl, 0.2 g KH_2_PO_4_, and 1.16 g Na_2_HPO_4_ were dissolved in 80 mL ddH_2_O, followed by mixing with 2 mL tween-20 and diluting into a 1 L solution). Then, the mixture was filtered through a 110 mm diameter glass microfiber filter (GE Whatman, Boston, MA, USA). An aliquot of 10 mL filtrate was passed through the immunoaffinity column (Hua’an Magnech Bio-Tech Co., Ltd., Beijing, China) and washed by 1 mL methanol into a glass tube. HPLC-FLD analysis was performed on a Waters 2695 Series LC system with a fluorescence detector (Ex = 274 nm, Em = 440 nm) (Waters Co., Ltd, Milford, MA, USA). Separation was conducted by a C_18_ column (5 µm particle size, 150 × 4.6 mm X-bridge, Waters Co., Ltd, Milford, MA, USA) at 25 °C and the injection volume was set as 10 μL. A mobile phase consisting of acetonitrile-water [50:50, v/v] was used at a flow rate of 1 mL/min.

#### 5.4.3. Analysis for the Distribution of Degradation Products 

To analyze the distribution of the degradation products, 1 mL of the water layer (b) and extraction of the oil layer (a) of each sample described above were passed through a 0.22 µm ultrafilter membrane (Waters, Milford, MA, USA) into glass tubes, separately. 

The analysis of the degradation products was performed on Agilent 6510 Quadrupole Time-of-Flight LC/MS (Agilent Technologies, Santa Clara, CA, USA) as Vekiru et al. described with slight modifications [24]. Briefly, the chromatographic column Agilent Poroshell 120 EC-C18 (2.1 × 100 mm, Agilent Technologies, Santa Clara, CA, USA) with a particle size of 2.7 µm. The injection volume was set as 10 µL. The mobile phase was a gradient prepared with A (0.1% formic acid and 5 mM NH_4_Ac aqueous solution) and B (0.1% formic acid methanol solution). The elution began with 3% B for 1 min, and the proportion of B was increased linearly to 45% at 5 min and to 98% at 16 min, kept for 2 min, then decreased back to 3% B at 18.5 min for 30 min. The flow rate of the mobile phase was controlled at 250 µL/min at 30 °C.

Typical parameters of the ESI source were as follows: ESI negative mode; drying gas temperature 325 °C, drying gas flow 10 L/min; nebulizer pressure 45 psi; capillary voltage 4000 V; fragmentor 140 V; skimmer 65 V; OCT1RFVpp 750 V. During the process, the spectrum acquisition rate was set at 2 spectra/second, data were collected within the range of 100 to 1200 m/z. To ensure the accuracy and reproducibility of the analysis, a lock spray was used to acquire accurate mass determination, and the Agilent reference mixture with two ions 112.9856 and 1033.9881 was used as well to calibrate the instrument. MassHunter B.07.00 software series (Agilent Technologies, Santa Clara, CA, USA) were used for further data acquisition and processing.

#### 5.4.4. Analysis of Tocopherols and Sterols

To evaluate the effect of enzymatic detoxification on the total tocopherol and sterol contents in corn oil, 50 g of each sample was used for the analysis of the total tocopherols following the protocols previously described by the national standard of China GB 5009.82-2016, and 250 mg of each sample was used to determine the sterol contents in accordance with the method described by the national standard of China GB/T 25223-2010.

### 5.5. Statistical Analysis

All of the treatments were carried out in triplicate. One-way analysis of variance was performed on all data. Statistical significance was determined by SPSS (version 19.0, SPSS, Inc., Chicago, IL, USA).

## Figures and Tables

**Table 1:** Nucleotide sequence of primers for constructing the recombinant plasmids.

Primer Name	Amplicon Gene	Primer Sequence (5’→3’)	Restriction Site
zlhy-6O-5’	zlhy-6O	CGGAATTC ATGAGAACTA GATCCACTAT	*Eco*R I
zlhy-6O-3’		ATAAGAATGCGGCCGCTTACAAGTACTTCTGAGTGA	*Not* I
GS- zlhy-6O-5’	GS+ zlhy-6O	CGCGGATCCATGTCTTTTAGATCCTTGTTGGCTTTGTCTGGTTTGGTTTGTTCTGGTTTGGCTATGAGAACTA GATCCACTAT	*Bam*H I
zlhy-6O-3’		ATAAGAATGCGGCCGCTTACAAGTACTTCTGAGTGA	*Not* I
IP-zlhy-6O-5’	IP+ zlhy-6O	CGCGGATCCATGAAGTTAGCATACTCCTTGTTGCTTCCATTGGCAGGAGTCAGTGCTATGAGAACTAGATCCACTAT	*Bam*H I
zlhy-6O-3’		ATAAGAATGCGGCCGCTTACAAGTACTTCTGAGTGA	*Not* I
IS-zlhy-6O-5’	IS+ zlhy-6O	CGCGGATCCATGCTTTTGCAAGCTTTCCTTTTCCTTTTGGCTGGTTTTGCAGCCAAAATATCTGCAATGAGAACTA GATCCACTAT	*Bam*H I
zlhy-6O-3’		ATAAGAATGCGGCCGC TTACAAGTACTTCTGAGTGA	*Not* I

Parts of the primer sequences highlighted in red are the sequences of glucoamylase signal (GS, Accession No. ABR58856.1), inulinase presequence (IP, Accession No. XP_022674031.1), and invertase signal (IS, Accession No. AAA35129.1). The underlined portion represents the restriction sites.
